# Chemotherapy in advanced bladder cancer: current status and future

**DOI:** 10.1186/1756-8722-4-35

**Published:** 2011-09-09

**Authors:** Nabil Ismaili, Mounia Amzerin, Aude Flechon

**Affiliations:** 1 Medical Oncology, Centre régional d'oncologie, Agadir, Morocco; 2 Medical Oncology, National institute of oncology, Rabat, Morocco; 3 Medical Oncology, Centre Léon-Bérard, Lyon, France

## Abstract

Bladder cancer occurs in the majority of cases in males. It represents the seventh most common cancer and the ninth most common cause of cancer deaths for men. Transitional cell carcinoma is the most predominant histological type. Bladder cancer is highly chemosensitive. In metastatic setting, chemotherapy based on cisplatin should be considered as standard treatment of choice for patients with good performance status (0-1) and good renal function-glomerular filtration rate (GFR) > 60 mL/min. The standard treatment is based on cisplatin chemotherapy regimens type MVAC, HD-MVAC, gemcitabine plus cisplatin (GC) or dose dense GC. In unfit patients, carboplatin based regimes; gemcitabine plus carboplatin or methotrexate plus carboplatin plus vinblastine (MCAVI) are reasonable options. The role of targeted therapies when used alone, or in combination with chemotherapy, or in maintenance, was evaluated; targeting angiogenesis seem to be very promising. The purpose of this literature review is to highlight the role of chemotherapy in the management of advanced transitional cell carcinoma of the bladder.

## 1- Introduction

The incidence of bladder cancer is increasing. An estimated 386,300 new cases and 150,200 deaths from bladder cancer occurred in 2008 worldwide [1]. The highest incidence is observed in Egypt with 37 cases per 100,000 inhabitants [2]. Bladder cancer occurs in the majority of cases in males with a male/female sex ratio of 3:1. It represents the seventh most common cancer for men [1]. In France, 10 700 new cases were diagnosed in 2000 and accounts for 3.5% of all cancer deaths. Bladder cancer is the sixth most common cancer (fifth most common cancer in men and seventh in women). In Morocco, bladder cancer was the sixth most common cancer in 2005 according to Rabat registry. The average age of diagnosis is 65 years [3].

Smoking is the most implicated risk factor in western countries, followed by other factors such as polycyclic aromatic hydrocarbons and cyclophosphamide [2]. In East Africa (especially Egypt), chronic infection with *Schistosoma haematobium *is the most common etiology and is often associated with squamous cell carcinoma [1,2].

Transitional cell carcinoma (TCC) is the most predominant histological type which represents more than 90% of the cases [4,5].

In more than 70% of the cases, the diagnosis is made at early stage of the disease (stages Ta and T1). Fifty percent of the patients with the disease at advanced stages (T2 or more) experience metastatic relapse.

In metastatic setting, chemotherapy treatment remains the only therapeutic option. It has the objective to alleviate the symptoms, to improve quality of life and to improve survival. In bladder TCC, chemotherapy showed very little progress and the standard MVAC is still the most used regimen and that since several years. New drugs are in the process of development, including those used in targeted therapies for which the role remains to be defined more clearly. This review emphasizes the role of chemotherapy and targeted therapies in metastatic bladder transitional cell carcinoma. Neoadjuvant or adjuvant chemotherapy, and systemic treatment of other histological types such as squamous cell carcinoma, adenocarcinoma, lymphoma, sarcoma and small cell carcinoma are not discussed in this article [4,5].

## 2- Methods of research

The literature review was conducted by using PUBMED data base using the following keywords: bladder cancer, transitional cell carcinoma, chemotherapy, cisplatin, and targeted therapies. The abstracts of papers presented at the annual meeting of the American Society of Medical Oncology (ASCO) were also analyzed. All Phase III trials were considered. The most important phase II trials have been also included in our article. The research was carried out from January 1980 until July 2011.

## 3- Prognostic factors in metastatic setting

Performance status (> 0), hemoglobin level (< 10 g/L), and liver metastasis are recognized as independent factors of poor prognosis in metastatic setting according to a recent prospective study. The median overall survival (OS) of 370 patients treated with chemotherapy for TCC carcinoma of the bladder with 0, 1, 2 and 3 factors were 14.2, 7.3, 3.8, and 1.7 months (P < 0.001), respectively [6].

Prognostic factors helps better to define the therapeutic strategy. For patients with 2 or 3 factors, it is suggested that aggressive chemotherapy should be avoided because of an increased risk of toxicity [6].

## 4- Chemotherapy in metastatic disease

### 4.1- Single agents

Bladder TCC are chemosensitive tumors. However, the response to a single agent is limited. Cisplatin is one of the most active drugs that give the highest overall response rate (ORR). Other drugs are also active (Table 1).

**Table 1:** ORR of single agents

Drogues	ORR
Cisplatin	33%
Methotrexate	29%
Doxorubicin	23%
5-fluoro-uracil	35%
Vinblastine	-
Cyclophosphamide	-
Mitomycine C	21%
Carboplatin	12-14%
Gemcitabine	24-28%
Paclitaxel	10-40%
Docetaxel	13-31%
Vinflunine	15%
Eribulin	38%

### 4.2- Multi agents chemotherapy

#### 4.2.1- Cisplatin-based chemotherapy

##### 4.2.1.1- Conventional regimens

The first protocols based on cisplatin (CMV: cisplatin, cyclophosphamide and vinblastine; and CISCA: cisplatin, doxorubicin and cyclophosphamide) induced 12 to78% ORR. The two protocols CMV and CISCA were widely used in the 1980s but did not show superiority in survival versus cisplatin alone [7-10].

Since 1990, the MVAC has been considered as a standard first-line therapy in metastatic disease. This regimen was for the first time studied in a nonrandomized phase II trial by Sternberg and colleagues in 1985 [11,12] and concerned 25 patients. They showed a sustained ORR in 71% of the cases and 50% of complete responses (CR). Two randomized phase III trials demonstrated the superiority of the MVAC to CISCA and CDDP, respectively, both in ORR, and in OS [13,14]. The MVAC is efficient, but particularly toxic. In the phase II study [11], the combination induced one toxic death and 4 febril neutropenias (16%), in addition to vomiting, anorexia, mucositis (grade 3-4 in 22% of the cases), alopecia and renal insufficiency.

To improve the results obtained with the MVAC, an intensification of this same protocol as HD-MVAC was tested in a phase III EORTC trial including more than 250 patients. In the experimental arm, all drugs were administered in day 1 and day 14. Prevention of toxicity was based on the routine use of Granulocyte Colony-Stimulating Factors (GCSF). Although the OS which represents the primary end point of the study, was identical in the two arms at 7.3 years median follow-up. However, the study showed that the intensification of the protocol improved CR (25 vs. 10%) and progression-free survival (PFS) (9.5 vs. 8.1 months, p = 0.03). Survivals at 2 and 5 years were also better (37% -22% vs. 25-22%, respectively). In addition, the systematic use of GCSF made the HD-MVAC better tolerated. While the primary end point was not achieved, the intensified MVAC is widely used in metastatic settings [15,16].

Table 2 summarizes the results of the most important phase III trials investigating first line chemotherapy in advanced bladder TCC.

**Table 2:** Randomized phase III trials investigating first-line chemotherapy regimens in metastatic bladder TCC

Authors	Journal or meeting	Year	Treatments	No	Results	Toxicity
Logothetis [13]	JCO	1990	MVAC vs. CISCA	120	Sup: ORR = 65 vs. 46%, p < 0.05, and OS = 62.6 vs. 48.3 weeks	Sup
Loehrer [14]	JCO	1992	MVAC vs. Cisplatin	146	Sup: ORR = 39 vs. 12%, p < 0.0001; PFS = 10 vs. 4.3 months, and OS = 12.5 vs. 8.2 months	Sup
Von der Maase [18,19]	JCO	2000	MVAC vs. GC	405	Equivalents	more neutropenic sepsis (12% vs. 1%; *P *< 0.001) and more grade 3-4 mucositis (22% vs. 1%; *P *= 0.001) on the MVAC arm
Sternberg [15,16]	JCO	2001	HD-MVAC vs. MVAC	259	Equivalents	less neutropenic fever (10% vs. 26%; *P *< 0.001) and mucositis on the HD-MVAC arm
Bamias [26]	JCO	2004	MVAC vs. DC	120	Sup	
Dreicer [31]	Cancer	2004	MVAC vs. PCa	85	Interrupted early	Sup
Bellmunt [48]	ASCO	2007	PCG vs. GC	627	Equivalents	Sup
De Santis [37]	ASCO	2010	GCa vs. MCAVI	238	Equivalents	Inf
Bamias [20]	ASCO	2011	DD-GC vs. HD-MVAC	175	Equivalents	Inf

Abbreviations. JCO: Journal of Clinical Oncology; MVAC: methotrexate-vinblastine-doxorubicin-cisplatin; CISCA: cisplatin-cyclophosphamide-doxorubicin; DC: docetaxel plus cisplatin; PCG: paclitaxel-cisplatin-gemcitabin; PCa: paclitaxel plus carboplatin; MCAVI: methotrexate-carboplatin-vinblastine; HD: high dose; DD: dose dense; ORR: objective response rate; OS: overall survival; PFS: progression free survival. Sup = superior; Inf = inferior.

##### 4.2.1.2- Second generation drugs

###### * Gemcitabine based regimens

In the 1990s, gemcitabine was a new molecule in the treatment of bladder TCC.

The first phase II trials evaluating the use of gemcitabine as single agent showed an improvement of ORR by 24 to 28%. The combination of gemcitabine with cisplatin (GC) has further improved these results with higher ORR (57%) and CR (15 to 21%) [17].

Based on these encouraging results, a phase III trial was conducted to compare the GC protocol to the standard MVAC. The study was designed to demonstrate superiority of the experimental arm in OS. The results showed no improvement of OS (MVAC: 14.8 months vs. GC: 13.8 months) and ORR (MVAC: 45.7 vs. GC: 49.4%). But due to the better safety profile, the GC was considered not inferior to MVAC [18,19].

A recent phase III trial compared the intensified HD-MVAC (n = 118) to the dense dose GC (DD-GC) (n = 57) (G: 2500 mg/m^2^, C: 70 mg/m^2 ^q 2 wks). The results were presented this year at the ASCO 2011 and showed that efficacy was similar in both treatments (ORR = 47.4 vs. 47.4%, respectively: p = 0.9; and OS = 18.4 vs. 20.7 months, respectively: p = 0.7), however, the safety profile was slightly better in favor to DD-GC [20].

###### *Taxanes based regimens

Cisplatin was also tested in phase II studies with other new drugs, particularly with taxanes (Table 3). The combination of cisplatin with paclitaxel and cisplatin with docetaxel improved ORR by 50-70% and 52-62% respectively [21-25].

**Table 3:** Phases II trials evaluating taxanes based doublets

Authors	Treatments	N	Results
Burch et al [21]	PC	34	ORR = 70%
Dreicer et al [22]	PC	52	ORR = 50% OS = 10.6 months
Dimopoulos et al [23]	DC	66	ORR = 52% TTP = 5 months OS = 8 months
DelMuro et al [24]	DC	38	ORR = 58% TTP = 6.9 months OS = 10.4 months.
Sengelov et al [25]	DC	25	ORR = 60% OS = 13.6 months

Abbreviations. PC: paclitaxel-cisplatin; DC: docetaxel-cisplatin; ORR: overall response rate; OS: overall survival; TTP: time to progression.

We note that these combinations remain inferior to the standard chemotherapy as was proven by the phase III randomized study conducted by the Hellenic Cooperative Oncology Group comparing docetaxel-cisplatin to MVAC. The standard protocol was superior in ORR (54.2% vs. 37.4%, p = 0.017), time to progression (TTP) (9.4 vs. 6.1 months, p = 0.003) and OS (14.2 vs. 9.3 months, p = 0.026) [26].

#### 4.2.2- Chemotherapy doublets based on other platinum drugs

Carboplatin is not as efficient as cisplatin. But has the advantage of being easily administered and better tolerated. Therefore, carboplatin-based protocols should be considered in patient ineligible (unfit) for cisplatin-based chemotherapy (Table 4) [27].

**Table 4:** Phases II trials evaluating carboplatin based doublets

Auteur	Treatment	No	Results
Redman et al [28]	PCa	35	ORR = 51%; OS = 9.5 months
Small et al [29]	PCa	29	ORR = 20,7%; TTP = 4 months; OS = 9 months
Vaughn et al [30]	PCa	33	ORR = 50%
Bellmunt et al [32]	GCa	16	ORR = 44%
Nogue-Aliguer et al [33]	GCa	41	ORR = 56.1%; PFS = 7.2 months; OS = 10.1 months
Shannon et al [34]	GCa	17	ORR = 58.8%; PFS = 4.6 months; OS = 10.5 mois
Dogliotti et al [35]	GCa vs. GC (Randomized phase II)	110	Efficacy: CR: 1.8% vs. 14.5%; OS: 9.8 vs. 12.8 months Toxicity: Equivalent.

Abbreviations. PCa: paclitaxel-carboplatin; GCa: gemcitabine-carboplatin; GC: gemcitabine-cisplatin; ORR: objective response rate; OS: overall survival; PFS: progression free survival

Carboplatin has been tested with paclitaxel in several phase II trials and permitted to achieve more than 63% ORR, but CR was limited as compared to cisplatin based protocols. Based on these frustrating results and other data suggesting the limited activity of this protocol [29,30], a phase III study was stopped early due to lack of recruitment. This study was designed to compare paclitaxel-carboplatin to MVAC [31].

Gemcitabine used in combination with carboplatin showed significantly lower results than cisplatin plus gemcitabine. ORR was high (59%), but the comparison with the GC showed that the standard arm was significantly better according to the results of one randomized phase II study [32-35].

Oxaliplatin is another platinum drug which showed only marginal activity as monotherapy [36].

In another hand, the EORTC conducted a phase III trial comparing unfit patients having metastatic TCC, the protocol based on carboplatin (AUC 4.5 on day) - gemcitabine (1000 mg/m^2 ^on day 1 and day 8) (GCa), repeated every 21 days, to the protocol M-CAVI [methotrexate (30 mg/m^2 ^on day 1, day 15, and day 22), carboplatin (AUC 4.5 on day 1) and vinblastine (3 mg/m^2 ^on day 1, day 15, and day 22)], repeated every 28 days. The results presented at ASCO 2010, confirmed the equivalence in OS between the 2 treatments, with a better toxicity profile in favor to the GCa protocol [37].

#### 4.2.3- Doublets without platinum drugs

Data on the effectiveness of drugs, in patients with good or poor condition are not sufficient. The literature reports only phase II trials with low number of patients. The protocol which is most studied is based on gemcitabine in combination with other molecules.

Gemcitabine-paclitaxel combination appears to produce a significant improvement. This protocol improved ORR to 40-60% [38-40]. Several schemes were tested. A phase II study investigated the gemcitabine-paclitaxel weekly, showed an ORR of up to 69% (42% of CR), however the rate of grade 3-4 pulmonary toxicity and toxic death is high. Therefore, the authors recommended disregard the use of this regimen in practice [41].

With docetaxel, gemcitabine is active and well tolerated. In 3 different phase II studies the ORR was between 30 and 50% [42-44].

Gemcitabine was also evaluated in association with pemetrexed in 2 phases II trials in 64 and 44 patients, respectively. The ORR was 20 and 28%. But this combination was very hematotoxic. In addition, 2 toxic deaths were reported [45,46].

#### 4.2.4- Triplets

To improve the ORR, several phase II and III studies were conducted by testing the addition of a third drug to the standard protocols used in practice.

Paclitaxel, in combination with GC, was the first triplet studied in a phase II trial conducted by Bellmunt, showing 77.6% ORR in 58 patients (ORR = 27.6% and PR = 50%) [47]. Therefore, the authors concluded the feasibility and the activity of this triple association. This was the background of a phase III randomized trial developed by the EORTC group, comparing the same protocol to the standard protocol GC. The authors considered the OS as a primary endpoint. Even with significant superiority in ORR for the experimental arm (57.1 vs. 46.4%, p = 0.02), the primary objective of the study was not achieved (OS = 15.7 vs. 12.8 months, p = 0.12, PFS = 8.4 vs. 7.7 months, p = 0.01) [48].

Bajorin has evaluated the feasibility and safety of paclitaxel, ifosfamide and cisplatin triplet administered every 3 weeks in a phase II study. Among 44 evaluable patients, the rate of CR was 23% and PR was 45%. The median survival was 20 months [49].

Paclitaxel-carboplatin-gemcitabine triplet was investigated in two phase II trials involving patients in the first line in one trial, and in 1st/2nd lines in another trial. ORRs and CR were equal to 43-68%, and 32-12%, respectively. The OS was equal to 14.7 and 11 months, respectively [50,51].

Other combinations including paclitaxel have also been reported in the literature, and showed promising activity and acceptable toxicity profile, but, more investigations are required in clinical trials [52-54].

The cisplatin-epirubicin-docetaxel triplet gave 30% complete responses in first line in 30 evaluable patients. The ORR was 66.7%. The median survival reached 14.5 months. Even for patients with PS 3, the overall safety profile was comparable to MVAC [55].

#### 4.2.5- Sequential protocols

Based on the effectiveness of the sequential regimens in breast cancer, this option was studied in metastatic bladder cancer.

In a phase II trial, the doublet doxorubicin-gemcitabine was evaluated in sequence with the triplet paclitaxel-ifosfamide-cisplatin in previously untreated patients (n = 60) with advanced TCC, with the systematic use of GCSF. In the final results recently published, the authors conclude that the regimen is active; however, it is associated with high rate of grade 3-4 hematological toxicity and does not clearly offer a benefit compared with the standard treatments [56]. In another trial, 25 patients with advanced urothelial carcinoma who were ineligible for cisplatin, received doses-dense sequential treatment with doxorubicin plus gemcitabine followed by paclitaxel plus carboplatin. ORR was 56% and the treatment was well tolerated [57].

Table 5 summarizes the most important prospective studies evaluating the role of triplet and sequential regimens.

**Table 5:** Phases I/II trials evaluated the triplets and sequential regimens

Authors	Treatments	Trial phase	No	Efficacy	Toxicity
Bellmunt et al [47]	CPG	I/II	58	ORR = 77.6%; CR = 27.6%; PR = 50%; OS = 24 months.	Hematological+++ (Grade 3/4 neutropenia and thrombocytopenia in 55% and 22%, respectively)
Bajorin et al [49]	ITP	II	44	CR = 23%.; PR = 45%.; OS = 20 months.	Well tolerated
Hussain et al [50]	CaPG	II	49	CR = 32%; PR = 36%; OS = 14.7 months; 1 years survival = 59%	Hematological+++
Hainsworth et al [51]	CaPG	II	60 (7% in 2nd line)	ORR = 43%; CR = 12%; OS = 11 months;	Hematological+++ (10% of febril neutropenia) One toxic death
Edelman et al [52]	M-CaP (GCSF)	I/II	33	ORR = 56%; OS = 15.5 months.	Hematological and neuropathy
Tu et al [53]	M-CP	II	25 (2^nd ^line)	PR = 40%; CR = 0%	Acceptable
Law et al [54]	M-GP	II	20	ORR = 45% (CR = 6; PR = 3); OS = 18 months; PFS = 6.3 months	Neutropenia+++ (1 toxic death)
Pectasides et al [55]	EDC	II	30	ORR = 66.7% (CR = 30%; PR = 36.7%); OS = 14.5 months.	Hematological (4 episodes of febril neutropenia)
Milowsky MI et al [56]	AG → ITP with GCSF	II	60	ORR = 73% (CR = 35% and PR = 38%); PFS = 12.1 months; OS = 16.4 months	Myelosupression (grade 3-4): 68% Febril neutropenia (25%)
Galsky MD et al [57]	AG → ITCa with GCSF	I/II	21	ORR = 56% (CR = 5; RP = 9)	Myelosupression (grade 3-4): 28% Febril neutropenia: 8%

Abbreviations. ITP: ifosfamide-paclitaxel-cisplatin; CPG: cisplatin-paclitaxel-gemcitabine; CaPG: carboplatin-paclitaxel-gemcitabine; M-CaP: methotrexate-carboplatin-paclitaxel; M-CP: methotexate-cisplatin-paclitaxel; M-GP: methotrexate-gemcitabine-paclitaxel; EDC: epirubicin-docetaxel-cisplatin; AG: doxorubicin-cisplatin; ITCa: ifosfamide-paclitaxel-carboplatin; CR: complete response; PR: partial response; ORR: objective response rate; OS: overall survival; PFS: progression free survival.

### 4.3- Second and third line chemotherapy

After failure of cisplatin-based first-line therapy, there was no consensus in the management of cisplatin resistant disease.

Taxanes (paclitaxel and docetaxel), vinflunine, and antifolate compounds (trimetrexate, piritrexim, and pemetrexed) resulted in 7 to 23% ORR. The FOLFOX4 was also studied in a phase II trial and resulted in 19% ORR [58-60]. In a recent published case study, the authors obtained a CR with FOLFOX4 chemotherapy in a metastatic urothelial cancer patient, after failure of GC combination [61].

The first phase III trial on cisplatin refractory setting compared vinflunine to best supportive care. Vinflunine is a semi-synthetic, vinca alkaloid compound that targets the microtubules. It was used at a dose of 320 mg/m^2 ^repeated every 21 days until progression or intolerance. Compared with the control arm, vinflunine was superior in OS > 2 months, however significant grade 3-4 hematologic toxicities (6% of febril neutropenia, one toxic death, anemia, and thrombocytopenia) were noted [62].

The second phase III trial was designed to compare a short-term (six cycles: arm A) versus prolonged (until progression: arm B) second-line combination chemotherapy of gepcitabine-paclitaxel. On prolonged treatment, more patients experienced severe anemia (arm A: 6.7% versus arm B: 26.7% grade 3-4 anemia; P = 0.011). Therefore, the authors concluded that it was not feasible to deliver a prolonged regimen. However, a high response rate of 40% makes the short protocol (6 cycles) a promising second line treatment option for patients with metastatic TCC [63].

### 4.4- New molecule

Eribulin is a new agent targeting the microtubules, being tested in several primary tumors. In advanced or metastatic TCC, this molecule was evaluated in a phase II trial, and showed a very interesting antitumor activity in front line with 38% ORR. The PFS was estimated to 3.9 months and the safety profile was acceptable (neutropenia, neuropathy, hypoglycemia, and hyponatremia) [64]. Based on these results, a phase III trial is undergoing to compare the standard GC to the combination of GC to Eribulin.

### 4.5- Targeted therapies

Despite the promising results obtained by chemotherapy based on MVAC or GC, the majority of patients die of metastatic disease.

The new progress in molecular biology has prompted the investigators to evaluate several molecules in metastatic bladder TCC.

**Figure 1:**
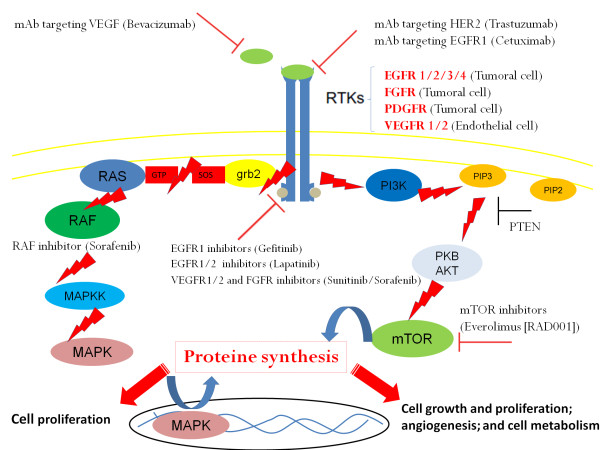
**Deregulated signaling pathways and targeted therapy in bladder cancer**. **Abbreviations: **EGFR, Epithelial Growth Factor Receptor; VEGFR, Vascular Endothelial Growth Factor R; FGFR: Fibroblast Growth Factor Receptor; mTOR: mammalian Target of Rapamycin.

Overexpression of several receptors such as the VEGFR (vascular endothelial growth factor receptor) on endothelial cells, the EGFR (epidermal growth factor receptor, the PDGFR (platlet derived growth factor receptor), and the FGFR (fibroblast growth factor receptor), on tumor cells, led the investigators to evaluate the efficacy and safety of new molecules targeting signaling pathways controlled by these proteins in metastatic setting (Figure 1).

The role of targeted therapy alone, in combination with chemotherapy, and in maintenance was evaluated using different molecules (bevacizumab, sunitinib, sorafenib, pazopanib, dovitinib, vandetanib, trastuzumab, cetuximab, erlotinib, lapatinib, everolimus, bortezomibe) (Table 6) [65-84].

**Table 6:** Phase II trials evaluating the role of targeted therapies

Organisations	Treatments	Trial phase	Line	No	Results	Most common grades 3-4 toxicities
Hoosier Oncology Group [66]	GC + Bevacizumab	II	1st	43	ORR = 72% (21% de; CR et 51% de RP); PFS = 8.2 months; OS = 20.4 months	Hematological, thromboembolism
USA (Texas) [70]	GC + sunutinib	II	1st	15	Interrupted for toxicity	Hematological++
Espagne [71]	Sunitinib	II	1st	37	DC = 8%; PFS = 5.9 months	Fatigue, Hypertention, Hand-Foot syndrom
Allemagne [73]	GC + sorafenib	IIR	1st	85	ORR = 82% vs. 78%; PFS = 6.3 mois vs. 7.2 months	Hematological
NCI Trial [79]	Trastuzumab + CaPG	II	1st	44 (HER2+++)	ORR = 70% (11% de CR et 59% de RP); OS = 14 months	Hematological, sensory neuropaty, cardiac
CALGB [81]	Gefitinib + GC	II	1st	58	ORR = 48%,; PFS = 7 months,; OS = 15 months; Equivalents results to GC et MVAC	Hematological, skin rash, diarrhea,
Allemagne [82]	Lapatinib	II	2nd and more	59	PR = 3%; S = 12%; PFS = 8.6 weeks	Diarrhea, vomiting, dehydration
Italy and USA [84]	Everolimus	II	2nd	45	PR = 8%; PFS = 3.3 months; OS = 10.5 months	Hematological, fatigue, metabolic, mucositis

Abbreviations. GC: gemcitabine-cisplatin; ORR: objective response rate; RC: complate response; PR: partial response; S: stabilisation; DC: disease control; PFS: progression free survival; OS: overall survival; CaPG: paclitaxel-gemcitabine-carboplatin.

#### 4.5.1- Targeting angiogenesis

Increased signaling through VEGFR and FGFR characterizes many TCC tumors and increased tumor vascularization. Angiogenesis is a very important step to tumor growth, invasion and metastasis. Therefore, targeting angiogenesis is a very interesting strategy which can be achieved by the use of monoclonal antibodies or by using small molecules tyrosine kinase inhibitors.

##### (A) Monoclonal antibodies

###### *Bevacizumab

Bevacizumab is a humanized monoclonal antibody (mAb) targeting the VEGF (Vascular Endothelial Factor) which has been approved by FDA in combination with chemotherapy as a standard treatment in first line and second line in different metastatic tumors. In bladder TCC, bevacizumab (15 mg/kg on day 1) was evaluated in first line treatment in combination with GC protocol (gemcitabine 1250 on D1 and D8 and cisplatin 70 mg/m^2 ^on D1, the cycle was repeated every 21 days) in a phase II trial (45 patients). Mature data presented at ASCO 2010 showed similar results in ORR and PFS to those obtained by the GC protocol, but OS was superior estimated to 20.4 months. A phase III trial comparing GC to GC plus bevacizumab is undergoing [66].

##### (B) Small molecules

###### *SU11248 Sunitinib Sutent ^®^

Sunitinib is a small molecule playing as a multi target intracellular tyrosine kinases inhibitor by inhibiting multiple receptors (EGFR, VERFR-1/2, C-KIT, PDGFR α/β) and the FLT3 and RET kinases. This drug has been approved by the FDA in the front line treatment of metastatic renal cell carcinoma and in the second line treatment of GIST (gastrointestinal stromal tumors) after failure of imatinib. Sunitinib has been tested in bladder cancer as single agent, in combination with chemotherapy, and in maintenance, and showed an interesting anti-tumor activity [67-71]. In a phase II trial presented at ASCO 2010, the Sunitinib was evaluated in association with the GC, but the trial was stopped because of high rate of hematological toxicity [70]. In another phase II study also presented at ASCO 2010, including 33 unfit patients treated with single agent sunitinib, the TTP was estimated to 4.8 months and the clinical benefit to 67%, confirming the role of the angiogenic pathway as an interesting target in the treatment of bladder TCC [71].

###### *BAY43-9006 Sorafenib Nexavar ^®^

Sorafenib is another small multi-target molecule (B-Raf, c-Raf, VEGFR-2/3, VEGFR-3, PDGFR-β) which has been approved by the FDA in second line treatment of metastatic renal cell carcinoma after failure of immunotherapy, and in first line treatment of advanced hepatocellular carcinoma, Child A. It has been tested in bladder TCC as single agent and in combination with chemotherapy in first and second line metastatic disease. However, Sorafenib didn't have activity in monotherapy [72], and in the combination with GC. Sorafenib did not improve the results of the standard GC in a recent randomized phase II trial [73].

###### *TKI258 Dovitinib

Dovitinib is an oral drug that inhibits angiogenic factors, including the FGFR and the VEGFR. TKI258, administered at a dose of 500 mg/day taken 5 days per week dosing schedule, was evaluated in phase II trial in second line treatment. The results of this trial, presented this year at the ASCO 2011, are promising [74].

#### 4.5.2- EGFR inhibitors

##### (A) Monoclonal antibodies

###### * Trastuzumab

The amplification of the HER2/neu oncogene has been correlated in bladder cancer to a more aggressive disease [75]. Bladder tumors with HER2 amplification represent 10-50% of cases [76-78].

In a multicenter U.S. Phase II study, trastuzumab was tested in combination with paclitaxel-carboplatin-gemcitabine triplet. The study included 109 patients, 57 (52%) had HER2 amplification, and 54 of 57 patients were treated with trastuzumab. The main toxicities were hematological, neurological and cardiac. ORR rate was equal to 70%. The TTP was 9.3 months and OS was14.1 months [79].

##### (B) Small molecules

###### * Gefetinib ZD1839 IRESSA ^®^

Gefitinib is a small molecule tyrosine kinase inhibitor that has been recently approved by the FDA in the front line treatment of metastatic non small cell lung cancer with activated EGFR mutation. In bladder cancer, it was in the first time evaluated as monotherapy in second-line therapy. This study showed no ORR. Median PFS was limited [80]. Gefitinib was also studied with GC in first line treatment. The results were similar to the GC and MVAC (CALGB 90102) [81].

###### * GW 572016 Lapatinib Tykerb ^®^

Lapatinib is a small molecule tyrosine kinase inhinitor allowing the inhibition of HER1 and HER2 receptors. This molecule has been approved by the FDA in the treatment of metastatic breast cancer with HER2 amplification. In one study, 59 patients with HER2 and/or EGFR amplifications were treated after failure of one or more therapeutic lines. In this phase 2 trial, only one patient (3%) had a partial response and 4 (12%) had stable disease [82].

#### 4.5.3- mTOR inhibitors

The mammalian target of rapamycin (mTOR) is an intracellular serine/threonine protein kinase positioned at a central point in a variety of cellular signaling cascades. The established involvement of mTOR activity in the cellular processes that contribute to the development and progression of cancer has identified mTOR as a major link in tumorigenesis. Consequently, inhibitors of mTOR, have been developed and assessed for their safety and efficacy in patients with cancer [83].

##### * Everolimus RAD001 AFINITOR ^®^

Everolimus is an oral rapamycin compound targeting and inhibiting the PI3K/Akt/mTOR pathway a central regulator of cell growth, proliferation, survival, and angiogenesis. It is currently indicated in second line treatment of metastatic renal cancer after failure of one tyrosine kinase inhibitor. The RAD001 was tested at a dose of 10 mg daily in 2 phase II trials in second line treatment. The results of these 2 trials were presented this year at ASCO 2011 and showed limited activity of Everolimus (PR = 8%; PFS = 3.3 months; OS = 10.5 months, in one study) [84].

#### 4.5.4- Histone deacetylase inhibitors

Histone deacetylases (HDACs) can regulate expression of tumor suppressor genes and activities of transcriptional factors involved in both cancer initiation and progression through alteration of either DNA or the structural components of chromatin. Recently, the role of gene repression through modulation such as acetylation in cancer patients has been clinically validated with several inhibitors of HDACs. In bladder cancer, Belinostat (PXD101) a HDACs inhibitor, was shown to be active according to several pre-clinical studies [85,86].

## 5- Treatment recommandations (Table 7)

### (A) First line treatment

In metastatic setting, chemotherapy based on cisplatin should be considered as standard treatment of choice for patients with good performance status (0-1) and good renal function-Glomerular filtration rate (GFR) > 60 mL/min. MVAC, HD-MVAC, gemcitabine-cisplatin and dose-dense gemcitabine-cisplatin should be considered as four standard first-line chemotherapy treatments for metastatic bladder TCC.

**Table 7:** Treatment recommendations:

First line treatment		Second and third line treatments	
**Patients eligible to cisplatin**	**Unfit patients**	**Cisplatin sensitive disease**	**Cisplatin refractory disease**

MVAC, HD-MVAC, GC, and DD-GC	GCa and MCAVI	Cisplatin based doublet not used in first line	Vinflunine, Paclitaxel-Gemcitabine, and all actives drugs not used

Taxane-based doublets are inferior to the standard MVAC and should not be used in first-line.

Carboplatin-based combinations are inferior to cisplatin based regimens and should be only used in unfit patients.

The platinum-free doublets are efficient and should be evaluated in randomized phase III trials.

The triplet combinations are more toxic but not more effective, and should not be used in practice.

The sequential protocols are more toxic but not more effective and should be evaluated in randomized phase III trials.

The role of targeted therapies in the management of metastatic bladder TCC has not yet been defined. Nevertheless, targeting angiogenesis seem to be very promising.

### (B) Second and third line treatments

For patients with platinum sensitive disease, a second line treatment based on cisplatin should be used in patient eligible to cisplatin. For cisplatin ineligible patients, a regimens based on carboplatin can be used.

Vinflunine and gemcitabine-paclitaxel are 2 reasonable therapeutic options in patients with cisplatin refractory disease.

All active drugs can be used in second and third line treatments.

## 6 - Conclusions

Chemotherapy plays a major role in the management of bladder cancer [87,88]. In the metastatic setting, palliative chemotherapy based on cisplatin type MVAC, HD-MVAC, or GC or DD-GC remains the treatment of choice. In unfit patients, Carboplatin based chemotherapy type Gemcitabin-Carboplatin or Methotrexate-Carboplatin-Vinblatine (MCAVI) is a good option for these patients. Novel therapies, targeting angiogenesis, have been shown to be very promising. Therapeutic investigations should be continued with the development of new drugs and targeted therapies to improve treatment results in the metastatic bladder cancer.

## Abbreviations

AKT: Protein Kinase B; ASCO: American society of clinical oncology; AUC: air under the curve; CaPG: carboplatin, Paclitaxel, and gemcitabine; CISCA: cisplatin, cyclophosphamide, and doxorubicin; C-Kit: Stem Cell Factor; CMV: cisplatin, methotrexate, and vinblastine; CR: complete response; DC: docetaxel plus cisplatin; DD-GC: dose dense GC; EDC: epirubicin, docetaxel, and cisplatin; EGFR: epidermal growth factor receptor; EORT: European organization of research and treatment; FDA: food and drug administration; FGFR: fibroblast growth factor receptor; GC: gemcitabine plus cisplatin; GCa: gemcitabine plus carboplatin; GCSF: granulocyte colony stimulating factor; GIST: gastrointestinal stromal tumor; HER2: human epidermal growth factor receptor 2; ITCa: ifosfamide, paclitaxel, and carboplatin; ITP: ifosfamide, paclitaxel, and cisplatin; MCAVI: methotrexate, carboplatin, and vinblastine; M-GP: methotrexate, gemcitabine, paclitaxel; mTOR: mammalian target of rapamycin; MVAC: methotrexate, vinblastine, doxorubicin, and cisplatin; HD-MVAC: high dose MVAC; ORR: overall response rate; OS: overall survival; PCa: paclitaxel plus carboplatin; PCG: paclitaxel, cisplatin, and gemcitabine; PDGFR: platelet derived growth factor; PFS: progression free survival; PI3K: phosphoinositide 3-kinases; PR: partial response; RAF: human proto-oncogene serine/threonine-protein kinase; S: stabilization; TCC: transitional cell carcinoma; TTP: time to progression VEGFR: vascular endothelial growth factor receptor

## Competing interests

The authors declare that they have no competing interests.

## Authors' contributions

NI is involved in concept design, in data collection, drafting and critically revising the manuscript. MA is involved in data collection; AF is involved in data collection and critically revising the manuscript.

All authors read and approved the final manuscript.
